# Predictors of early faculty attrition at one Academic Medical Center

**DOI:** 10.1186/1472-6920-14-27

**Published:** 2014-02-10

**Authors:** Brenda A Bucklin, Morgan Valley, Cheryl Welch, Zung Vu Tran, Steven R Lowenstein

**Affiliations:** 1 Department of Anesthesiology and Office of Undergraduate Medical Education, University of Colorado School of Medicine, Aurora, Colorado, USA; 2 Fort Collins, Colorado, USA; 3 Office of Faculty Affairs, University of Colorado School of Medicine, Aurora, Colorado, USA; 4 Department of Biostatistics and Informatics, Colorado School of Public Health, Aurora, Colorado, USA; 5 Department of Emergency Medicine and Medicine, Office of Faculty Affairs, University of Colorado School of Medicine and Colorado School of Public Health, Aurora, Colorado, USA

## Abstract

**Background:**

Faculty turnover threatens the research, teaching and clinical missions of medical schools. We measured early attrition among newly-hired medical school faculty and identified personal and institutional factors associated with early attrition.

**Methods:**

This retrospective cohort study identified faculty hired during the 2005–2006 academic year at one school. Three-year attrition rates were measured. A 40-question electronic survey measured demographics, career satisfaction, faculty responsibilities, institutional/departmental support, and reasons for resignation. Odds ratios (ORs) and 95 percent confidence intervals (95% CI) identified variables associated with early attrition.

**Results:**

Of 139 faculty, 34% (95% CI = 26-42%) resigned within three years of hire. Attrition was associated with: perceived failure of the Department Chair to foster a climate of teaching, research, and service (OR = 6.03; 95% CI: 1.84, 19.69), inclusiveness, respect, and open communication (OR = 3.21; 95% CI: 1.04, 9.98). Lack of professional development of the faculty member (OR = 3.84; 95% CI: 1.25, 11.81); institutional recognition and support for excellence in teaching (OR = 2.96; 95% CI: 0.78, 11.19) and clinical care (OR = 3.87; 95% CI: 1.04, 14.41); and >50% of professional time devoted to patient care (OR = 3.93; 95% CI: 1.29, 11.93) predicted attrition. Gender, race, ethnicity, academic degree, department type and tenure status did not predict early attrition. Of still-active faculty, an additional 27 (48.2%, 95% CI: 35.8, 61.0) reported considering resignation within the 5 years.

**Conclusions:**

In this pilot study, one-third of new faculty resigned within 3 years of hire. Greater awareness of predictors of early attrition may help schools identify threats to faculty career satisfaction and retention.

## Background

Several large studies have examined the turnover of faculty at U.S. medical schools [1-3]. According to some estimates, 5 of every 10 clinical faculty members leave their medical school appointments within 10 years, and as many as 4 out of 10 leave academic medicine entirely [1]. Studies suggest that faculty leave academic medicine for a variety of reasons, including business and revenue pressures [4-6], burdensome regulations [7,8], lack of recognition for teaching or clinical excellence [9], lack of academic mentorship and difficulties balancing family and work [10,11]. Whatever the reasons, faculty attrition represents a serious loss of human and financial capital that may threaten the research, teaching and clinical service missions of institutions [12-14].

Faculty attrition is likely to become even more problematic in the future. An aging physician workforce [15,16], increased numbers of part-time physicians, and younger physicians who view work-life balance as essential [17] will pressure academic medical centers to recruit and retain talented clinical faculty members. In addition, physician workforce shortages of more than 90,000 are expected in the next decade [15]. These changes will increase competition for physicians being recruited from the private sector and challenge academic medical centers as they struggle with recruitment and retention.

Although several studies have examined faculty satisfaction and turnover at U.S. medical schools, few studies have specifically examined attrition among new faculty members who leave their posts shortly after being hired. Therefore, we conducted the current study in order to learn more about faculty members who leave their medical school posts within three years of hire (“early attrition”). Specifically, we sought to measure the rate of early attrition in a cohort of new faculty hires and identify demographic, job-related and institutional factors that were associated with early attrition.

## Methods

### Setting

The University of Colorado School of Medicine (UCSOM) is a public medical school with more than 2,700 full-time faculty members who are engaged in the missions of teaching, research and clinical and community service. Faculty members are employed by the University or by one of several affiliated hospitals.

### Selection of study participants

We utilized the UCSOM electronic faculty database to identify a cohort of faculty at all faculty ranks with terminal degrees (MD, DO, PhD or combinations of these). These faculty members were hired during a single academic year (2005–2006). We determined the employment status of each faculty member (resigned or still active) after the three-year follow-up period (2009).

### Survey of faculty characteristics, attitudes and experiences

In 2010 (approximately five years after the original date of hire), we distributed a 40-question, web-based and anonymous survey to members of the faculty cohort after Colorado Multiple Institutional Review Board (COMIRB Protocol 10–0444; University of Colorado Denver, Anschutz Medical Campus) approval. We distributed the survey to all faculty members for whom contact information was available, whether they were still active faculty members or had resigned. Survey questions addressed demographic characteristics (gender, race and ethnicity), academic rank, highest degree, departmental affiliation, tenure status and whether the faculty member was (or had been) a member of a multi-disciplinary center. Participants were asked to indicate the “percentage of your total work time that you spend each week, on average, in each of the following areas (education, research and scholarship, patient care, administration or other”). Participants were also queried about the adequacy of institutional and departmental support for teaching, clinical work and research. For each question measuring faculty experiences, faculty members were asked whether they agreed or disagreed with a statement (for example, “There is a sense of academic community and collaboration in my department, section or division”). Likert scales were used to measure agreement (“strongly agree”, “agree”, “disagree” or “strongly disagree”). For clarity and ease of analysis, these ordinal responses were later collapsed into dichotomous categories: “agree” (including “strongly agree” and “agree”) or “disagree” (“disagree” or “strongly disagree”).

The survey also included open-ended questions asking for examples or more detailed information about various topics. Participants’ free-text responses were not analyzed qualitatively, but comments were reviewed for consistency with the results of the quantitative analysis, and for illustrative examples.

Using a multiple select option, faculty who had resigned were asked “What primary professional or personal consideration impacted your decision to leave the University of Colorado School of Medicine?” Faculty members who were still active were asked, “Are you considering leaving the School of Medicine in the next five years?” In addition, these faculty members were asked about their reasons for considering resignation.

### Data analysis

The rate of faculty attrition was measured over a 3-year period (2006–09). The analysis of the data then proceeded in two steps. First, for measurement variables, all demographic and job-related characteristics and survey responses were summarized using means and standard deviations, or medians and ranges. Proportions and 95% confidence intervals (95% CI) were used for categorical variables. Second, bivariate analyses were performed to test for associations between the principal outcome (employment status) and each covariate. To measure the strength of the associations between each of these variables and employment status, odds ratios (ORs) and 95% confidence intervals (95% CI) were calculated.

## Results

### Sample characteristics

One hundred thirty-nine faculty members with doctoral degrees were hired in academic year 2005–06. Five years later (2010), when the survey was administered, 92 faculty members (66.2%, 95% CI: 57.6-73.9%) were still employed at the CUSOM and 47 (33.8%, 95% CI: 26.2-42.4%) had resigned. (see Table 1). Of the 47 faculty members who had resigned, 41 (87%) were instructors or assistant professors. Thirty-seven (78%) of the 47 faculty members who resigned were MDs or DOs.

**Table 1:** Demographics of the study population (faculty cohort hired in 2005–06 at the University of Colorado School of Medicine)

	**Total faculty hires (n)**	**Total survey respondents% (n)**	**Active survey respondents% (n)**	**Survey respondents who had resigned% (n)**
Highest Degree				
MD or DO	72% (100)	65% (50)	62% (37)	77% (13)
MD/PhD	6% (9)	12% (9)	15% (9)	0
PhD	22% (30)	23% (18)	23% (14)	23% (4)
Rank				
Instructor	41% (57)	33% (25)	29% (17)	47% (8)
Assistant Professor	38% (53)	45% (34)	42% (25)	53% (9)
Associate Professor	8% (11)	9% (7)	12% (7)	0
Professor	13% (18)	13% (10)	17% (10)	0
Gender				
Male	58% (81)	58% (44)	59% (35)	53% (9)
Female	42% (58)	42% (32)	41% (24)	47% (8)
Race/Ethnicity				
Asian	11% (15)	15% (11)	14% (8)	18% (3)
African American	<1% (1)	0	0	0
Hispanic	3% (4)	5% (4)	5% (3)	6% (1)
Native American	<1% (1)	1% (1)	2% (1)	0
Caucasian	85% (118)	79% (60)	80% (47)	77% (13)
Employment Status				
Active	66% (92)	65% (60)		
Resigned	34% (47)	36% (17)		
Total	139	100% (77)		

Among the still-active faculty members, 60 (65%) participated in the survey; the participation rate was lower among faculty members who had resigned (17 of 47; 36%). Active and non-active faculty members who completed the survey did not differ significantly with respect to gender (p = 0.64), race (p = 0.93), Hispanic ethnicity (p = 0.90), type of highest degree earned (p = 0.22), department type (clinical or basic science, p = 0.21) tenure status at time of hire (p = 0.35), membership in a clinical or research center (p = 0.87), or percentage of time devoted to research (p = 0.47). One-hundred percent of non-active faculty who participated in the survey were at the rank of Instructor or Assistant Professor compared to 70% of active faculty at the same ranks (p = 0.09). (see Table 1).

### Nature of work

Table 2 presents the results of the survey of nature of work. There were no significant differences between active and inactive faculty members with regard to time spent in educational, administrative, research and scholarly activities. However, faculty members who spent more than 50 percent of their time in direct patient care were more than 3 times more likely to have resigned (OR = 3.93; 95% CI: 1.29, 11.93). Fifteen survey respondents commented on the extensive clinical commitment of faculty members. One faculty member who resigned stated, “I paid the price for my own success, building a program and caring for a large number of patients. I was hindered by the clinical workload, working 200% rather than 100%”. Another faculty member who resigned said, “The focus was on providing care for patients. Academic progress was something that had to be done on my own time”.

**Table 2:** Nature of work of survey participants

**More than half of time spent on:**	**Total faculty% (n)**	**Active faculty% (n)**	**Faculty who had resigned% (n)**	**OR (95% CI)**
Educational Activities	11% (8)	5% (3)	29% (5)	7.78 (1.63, 37.06)
Research and Scholarly Activities	23% (17)	24% (14)	19% (3)	0.74 (0.18, 2.98)
Patient Care Services	37% (27)	29% (16)	61% (11)	3.93 (1.29, 11.93)
Administration	8% (6)	9% (5)	6% (1)	0.62 (0.07, 5.71)
Other	7% (3)	0% (0)	9% (3)	P = 0.327*

*p-values from Fisher’s Exact Test are listed when OR could not be calculated due to 0 in cells.

### Predictors of early faculty attrition

Table 3 shows the variables associated with resignation within three years of hire, with respect to institutional and departmental climate, culture and collegiality, faculty development, career progression, mentoring, feedback, and rewards. Faculty members who resigned were more than six times more likely to report that their Department Chair had failed to establish a scholarly environment (OR 6.03; 95% CI: 1.84, 19.69). Other variables associated with attrition were the perceived lack of interest by the Chair in the faculty member’s professional development (OR 3.84; 95% CI: 1.25, 11.81) and lack of a departmental climate of inclusiveness, respect, and open communication (OR 3.21; 95% CI: 1.04, 9.98). Typical comments from survey respondents who resigned were, “As a sub-specialist, I felt that our chairperson didn’t care about me (i.e., you don’t bill enough for me to care about you). The hospital took advantage of my work. There were demands for caring for many difficult and after-hours patients. I wasn’t compensated in any way.” Others said: “The Chairman didn’t care about me or my academic progress;” “they promised me things in regard to career development and they never delivered;” and, “I took the position thinking the Chair was going to be a personal mentor. Instead, I felt only betrayal and non-interest from him”. Faculty attrition was also associated with lack of institutional recognition and support for excellence in teaching (OR 2.96; 95% CI: 0.78, 11.19) and clinical care (OR 3.87; 95% CI: 1.04, 14.41).

**Table 3:** Factors associated with early faculty attrition at the University of Colorado School of Medicine

	**Active faculty% (n)**	**Faculty who had resigned% (n)**	**OR (95% CI)**
Department Chair does not establish an environment that fosters teaching, research, creativity, and service to the School and community	21.7% (13)	62.5% (10)	6.03 (1.84, 19.69)
More than 50% of time devoted to patient care	28.6% (16)	61.1% (11)	3.93 (1.29, 11.93)
Department Chair (section or division head) is not interested in my professional development	26.7% (16)	58.8% (10)	3.84 (1.25, 11.81)
Department does not foster a climate of inclusiveness, respect, and open communication	21.7% (13)	47.1% (8)	3.21 (1.04, 9.98)
School of Medicine does not reward excellence in clinical service	39.5% (17)	69.2% (9)	3.87 (1.04, 14.41)

### Professional and personal considerations impacting the decision to resign

Among all faculty members who resigned, only 11.8% (95% CI: 3.3, 34.3%) took a position at another university; 52.9% (95% CI: 31.0, 73.8%) left for work in private practice or a non-profit group practice. The most common reasons for leaving included: career not progressing at a satisfactory pace (29.4% (95% CI: 13.3, 53.1%); inadequate salary support (23.5%, 95% CI: 9.6, 47.3%); dissatisfaction in the University of Colorado as a place to work (17.6%, 95% CI: 6.2, 41.0%); and better opportunities elsewhere for promotion or career advancement (17.6%, 95% CI: 6.2, 41.0%).

### Current faculty members considering resignation

Among the still-active faculty who completed the survey, 27 (48.2%, 95% CI: 35.7, 61.0%) stated that they were considering resigning within the next five years. Reasons for considering resignation included: inadequate salary support (33.3%, 95% CI: 18.6, 52.2%); dissatisfaction with the School of Medicine as a workplace (25.9%, 95% CI: 13.2, 44.7%); lack of outside funding (22.2%, 95% CI: 10.6, 40.8%); unsatisfactory pace of career advancement (18.5%, 95% CI: 8.2, 36.7%); and lack of feedback from department leadership (14.8%, 95% CI: 5.9, 32.5%).

## Discussion

This study was conducted in a single public medical school and is one of the first to focus solely on new faculty hires. Consistent with a growing number of studies of medical school faculty evaluating turnover and career satisfaction, results of this study demonstrate that more than one-third of newly-hired faculty members left the institution within 3 years of hire. In addition, nearly 50% of the still-active faculty members are considering leaving in the next 5 years. Besides evaluation of actual rates of early faculty attrition and intent to leave, this report examines why faculty were dissatisfied and left our institution or academic medicine altogether. In the study, clinicians and junior faculty members left at high rates. Predictors of early attrition included more than 50% of professional time devoted to direct patient care, perceived lack of interest by the department chair in the faculty member’s professional development, lack of feedback by the chair or supervisor regarding academic progress, absence of an “academic community”, and lack of institutional recognition and support for excellence in teaching and clinical care. Gender, race, ethnicity, academic degree, department type, tenure status or percentage time devoted to research did not predict early attrition. Although these rates of attrition and factors associated with attrition are consistent with previous studies [1,3,13,14], early attrition may be an indicator of early faculty dissatisfaction and misaligned expectations rather than academic success or promotion which often leads to opportunities at other academic medical centers.

Faculty turnover threatens the research, teaching, and clinical missions of academic medical centers who are challenged by the loss of human and financial capital associated with these losses. Although there has been increasing interest in faculty attrition, this issue will assume even greater importance because of the financial impact of implementation of value-based health care and reduction of the bottom line. Further limits on financial resources, a need to lower health care costs, and to improve quality and outcomes will increase pressure on already stressed academic medical center faculty [11].

Due to the substantial institutional resources that are used for recruitment [18,19], early loses may represent an even greater negative return on the institutional investment. Estimates suggest that it takes between two and four years for faculty members to generate revenues that exceed recruitment costs (i.e., start-up package, institutional support, time to develop a patient referral base) [19]. Although our study did not measure the human and financial cost of attrition in the cohort, loss of clinicians is expensive with estimates ranging from $115,554 to replace a generalist and $587,125 to replace a surgical subspecialist [13]. The loss of basic scientists also has a large impact on the finances of academic medical centers. One medical school estimated that over a 7-year period, the cost to support a cohort of new faculty hires in basic science was a total of $69.0 million ($33.1 million in start-up costs and $35.9 million in indirect costs) [12]. Although the faculty generated $1.45 in total grant revenue for every dollar invested, start-up costs and incomplete recovery of indirect costs required the school to add 40 cents for every dollar generated to achieve financial equilibrium. Decreased morale, increased disruption, lost teaching opportunities, unsuccessful searches, recruitment costs, decreased productivity in the months prior to departure and during the time until a newly recruited faculty member becomes fully functional are intangible costs that all contribute to the cost of faculty attrition. “Academic medical centers need to be concerned about attracting the next generation of faculty and leaders as well as losing productive faculty who are disenchanted and struggling in the current academic setting” [11].

Current levels of medical school faculty satisfaction are reported to be somewhat lower than previous reports [20]. However, reasons for faculty dissatisfaction and discontent are multifaceted and often generalized. Previous studies suggest that an emphasis on department and institutional relationships, collegiality, academic community [11,21], faculty engagement [22], regular feedback and recognition for excellence in teaching and clinical service, effective retention and recruitment [11], equality of institutional missions [23], transparency, effective institutional and departmental leadership [24,25], workplace culture [22], and nature of work are important contributors to faculty satisfaction. Our study results are consistent with other reports of faculty dissatisfaction, attrition, and intent to leave in that workplace culture, departmental relationships, professional development opportunities, and nature of work are important contributors to faculty satisfaction.

Several reports have evaluated the reasons for faculty dissatisfaction and intent to leave their institution or academic medicine entirely. Predictors of intent to leave were feelings of vulnerability and lack of connection with colleagues, moral distress, and being “adversely changed” by the academic culture [22]. Of concern are also high rates of depression and anxiety in both men and women at four medical schools, although the authors stated that it was unclear how mental health affects satisfaction and attrition [2]. Because the consequences of faculty discontent and attrition have widespread effects, the effects of dissatisfaction extend beyond faculty members themselves. It is well-known that patient satisfaction and quality of care are positively correlated with physician job satisfaction [26,27]. In addition, an unfavorable medical school culture impacts the hidden curriculum for medical students resulting in increased cynicism and reduced altruism among students [28,29]. Because of the wide range of effects, it will be important for academic medical centers to monitor workplace satisfaction, especially with a multigenerational workplace, projected workforce shortages, and a changing healthcare environment in order to retain and recruit faculty members.

Recommendations to promote recruitment and retention as well as limit faculty dissatisfaction and discontent are abundant. Given the institutional cost of faculty attrition [18,19], especially costs related to attrition of junior faculty members [13,18], setting clear expectations at the time of hiring and identifying faculty whom the institution would like to retain would be first steps in cost containment. Institutions and departments should regularly assess rates of attrition to create greater awareness and implement programs to effectively monitor, understand, and improve the culture in academic medical centers. Our study is consistent in that departmental leaders play an important role in faculty satisfaction by promoting a culture of transparency, open communication, inclusiveness, and opportunities [11,25]. Reports suggest that institutional leaders and chairpersons are instrumental in creating the culture. As leaders, they must recognize talent, assist in developing an academic career path, provide feedback, and set clear expectations about job performance. The leadership must also recognize the importance of equal valuation of missions as an important contributor to faculty dissatisfaction. This is consistent with our study in that attrition was associated with a lack of recognition for excellence in teaching or clinical care. Studies also suggest that faculty development and mentoring programs have an important impact on faculty retention and success in academic medicine. More recently, sponsorship has emerged as a way to advance promising talent in academic medicine [30]. As academic medical centers face challenges with health-care reform, physician shortages, and the need to recruit and retain faculty, they should monitor and develop programs to ensure faculty satisfaction. Such programs would be more cost-effective than constant recruitment and the time it takes to generate revenues that exceed recruitment costs.

It is important to recognize several limitations of this study. First, the data were collected from a single cohort of mostly junior faculty members from a single, public medical school. The majority of the newly-hired faculty members were Caucasian, and many were clinical track faculty members. Among faculty members who left our institution, only 36% returned the survey, raising the possibility of non-participation bias. Faculty members who did not return their surveys may have had higher, or lower, levels of dissatisfaction with the School of Medicine or different reasons for resigning. Future analyses could be improved by collection of data from multiple years and institutions to provide a broader, more longitudinal perspective.

Faculty members are essential human resources of academic medical centers. Some attrition is inevitable and is likely beneficial, providing an opportunity for recruitment of new faculty with novel and innovative ideas. However, increased demands for generation of clinical income make it more difficult for some faculty members to participate in academic work and the teaching and research missions of a medical school.

## Conclusions

Because substantial institutional resources are used for recruitment, and early losses may represent an even greater negative return on the institutional investment, institutions should regularly assess rates of attrition to create greater awareness and implement timely measures to reduce them. As the physician workforce becomes increasingly multigenerational, it will be important to align expectations and determine how to best recruit, integrate and support these faculty members in their new roles.

## Competing interests

The authors declare that they have no competing interests.

## Authors’ contributions

BB conceived of the study, participated in the design of the study, and drafted the manuscript. MV participated in the design of the study and performed the statistical analysis. CW participated in the design and coordination of the study. ZT consulted on statistical analysis. SL participated in the design of the study and helped draft the manuscript. All authors read and approved the final manuscript.

## Pre-publication history

The pre-publication history for this paper can be accessed here:

http://www.biomedcentral.com/1472-6920/14/27/prepub
